# Functional Hybrid Materials Based on Manganese Dioxide and Lignin Activated by Ionic Liquids and Their Application in the Production of Lithium Ion Batteries

**DOI:** 10.3390/ijms18071509

**Published:** 2017-07-12

**Authors:** Łukasz Klapiszewski, Tadeusz J. Szalaty, Beata Kurc, Małgorzata Stanisz, Andrzej Skrzypczak, Teofil Jesionowski

**Affiliations:** 1 Institute of Chemical Technology and Engineering, Faculty of Chemical Technology, Poznan University of Technology, Berdychowo 4, PL-60965 Poznan, Poland; tadeusz.h.szalaty@doctorate.put.poznan.pl (T.J.S.); malgorzata.stanisz@student.put.poznan.pl (M.S.); andrzej.skrzypczak@put.poznan.pl (A.S.); teofil.jesionowski@put.poznan.pl (T.J.); 2 Institute of Chemistry and Technical Electrochemistry, Faculty of Chemical Technology, Poznan University of Technology, Berdychowo 4, PL-60965 Poznan, Poland; beata.kurc@put.poznan.pl

**Keywords:** kraft lignin, ionic liquids, manganese oxide, physicochemical and structural characteristics, electrochemical properties

## Abstract

Kraft lignin (KL) was activated using selected ionic liquids (ILs). The activated form of the biopolymer, due to the presence of carbonyl groups, can be used in electrochemical tests. To increase the application potential of the system in electrochemistry, activated lignin forms were combined with manganese dioxide, and the most important physicochemical and morphological-microstructural properties of the novel, functional hybrid systems were determined using Fourier transform infrared spectroscopy (FTIR), elemental analysis (EA), scanning electron microscopy (SEM), zeta potential analysis, thermal stability (TGA/DTG) and porous structure analysis. An investigation was also made of the practical application of the hybrid materials in the production of lithium ion batteries. The capacity of the anode (MnO_2_/activated lignin), working at a low current regime of 50 mA·g^−1^, was ca. 610 mAh·g^−1^, while a current of 1000 mA·g^−1^ resulted in a capacity of 570 mAh·g^−1^. Superior cyclic stability and rate capability indicate that this may be a promising electrode material for use in high-performance lithium ion batteries.

## 1. Introduction

Lignin is a naturally occurring polymer that can be found in wood and plants [1,2,3]. It protects cells against biological attack, controls water transport and increases mechanical strength [3]. This biopolymer can be synthesized through oxidative coupling of three main monomers, derivatives of phenolic alcohols: *p*-coumaryl, synapil and coniferyl alcohols [1,2,3,4,5]. The surface of lignin is rich in a variety of functional groups such as hydroxyl, carboxyl and ether groups. The presence of these functional groups allows selective modification of this complex compound [3].

Lignin is a byproduct in the paper and pulp industry, and has been used chiefly as a fuel to provide heat for the technological processes [6]. Lignin is the only source for the production of renewable aromatic compounds [7]. It can also be used as an adsorbent of dyes [8] and heavy metal ions [9], as a cathode in simple lithium batteries [10], and in the synthesis of polyethers, polyurethanes [5] and epoxy resins [11]. A very innovative approach is to synthesize a hybrid material of silica and lignin and to use it as a sorbent for metal ion removal [12], as a polymer filler [13,14,15] and as a component for abrasive tools [16]. Lignin can be used in its untreated state. However, in order to fully exploit its properties, its natural conformation needs to be improved by means of certain structural modifications.

Activation of the biopolymer is carried out to modify functional groups while leaving the aromatic base of the polymer intact [17]. Originally, lignin was subjected to oxidation to better understand its structure and to identify its bonds. At present, it is known that the use of strong oxidants breaks up the aromatic ring of the polymer, whereas milder ones change only its functional groups [18]. The lignin surface contains many hydroxyl groups, which can be oxidized to carbonyl groups. These are more reactive than the hydroxyl groups, and so the resulting compound may offer more potential applications in electrochemistry [3].

A large number of approaches to the activation of lignin have been investigated. Oxidation can be conducted using various chemical oxidants. Catalysts such as TiO_2_ may be used with UV light to initiate the process [19] and simple metal salts CuSO_4_ and FeCl_3_ can be used to produce aromatic ketones and aldehydes from lignin [20]. Other compounds employed to activate the biopolymer include KMnO_4_, NaIO_4_ [21,22] and H_2_O_2_ [23]. In alkaline conditions and with O_2_ as an oxidant, metal complexes are used, e.g., Co(salen), Co(sulfosalen) and Co(*N*,*N*′-bis(acetylacetone)ethylenediamine) [24]. High oxidation reactivity is demonstrated by enzymes such as laccase [25] and Mn-peroxidases (MnP) [26].

One of the latest oxidation methods for lignin relies on the use of ionic liquids (ILs) as solvents [27,28,29,30,31,32,33,34]. ILs have unique properties, including no detectable vapor pressure, low melting point and good thermal and chemical stability [35,36,37]. The oxidation of lignin in ionic liquids can be performed at low temperatures and pressures, and after the process the catalyst can be separated from the product and used again, which is advantageous in terms of green chemistry and low waste production [38].

Lignin can be oxidized in ionic liquids with the addition of metal catalysts such as Fe, Mn and V and oxidizing agents such as O_2_ and H_2_O_2_ [39]. Wiermans et al. studied the importance of H_2_O_2_ concentration during lignin activation in ionic liquids. Their results showed that 5% H_2_O_2_ oxidized lignin and its structure remained non-degraded. However, adding 10% H_2_O_2_ resulted in lignin molecule depolymerization and oxidation [40]. Prado et al. depolymerized lignin in two different ionic liquids (butylimidazolium hydrogen sulfate [HC_4_im][HSO_4_] and triethylammonium hydrogen sulfate [Et_3_NH][HSO_4_]) with H_2_O_2_ as oxidizing agent. It was observed that adding a higher contraction of oxidant resulted in phenolic derived compounds such as vanillin acid [39]. It is noteworthy that reaction conditions are crucial for the lignin oxidation process. Studies have shown that using more acidic ionic liquids results in an efficient lignin activation process [41]. Shiwei et al. activated lignin using four dimethyl phosphate-based ILs. The process was catalyzed by CuSO_4_ with O_2_ as an oxidizing agent. The pH of the reaction environment was lower than that of NaOH. It was found that in weaker alkaline conditions, the activity of the oxygen increased [42]. Brandt et al. separated lignin from biomass using a mixture of protic ionic liquid 1-butylimidazolium hydrogen sulfate [HC_4_im][HSO_4_] with water. Their work showed that during ionic liquid pretreatment lignin-hemicellulose linkages were broken and lignin depolymerized, resulting in smaller molecular compounds. When lignin was pretreated for longer, repolymerization through condensation reactions was observed [43].

Recently, hybrid materials have been developed to provide improved or new unique properties. Compounds used to create hybrid materials include manganese oxides such as MnO or MnO_2_. Manganese oxides are characterized as nontoxic and efficient catalytic materials that are easy to synthesize [44]. Manganese oxides are used most widely as electro design batteries [45], electrochemical pseudocapacitors [46,47] and electrochemical cells [48]. Among the attractive anode materials, manganese dioxide has emerged as an effective alternative to graphite anodes because it offers an extremely high theoretical specific capacity of 1230 mAh·g^−1^ and excellent electrochemical behavior, alongside its low price, natural abundance and environmental friendliness [49,50,51], but it suffers from significant capacity loss and poor cyclic stability due to the large volume expansion during cycling, which limit its widespread application [50,52]. To meet such challenges, tremendous work has been focused on the most popular method of building nanostructured materials.

The capacitance of manganese oxide electrodes is limited because of its poor electrical conductivity [53]. To enhance the electrical conductivity and charge-storage capability of manganese oxide electrodes, modifications have to be carried out—for instance, adding to the electrode other transition metal elements such as Ni, Cu and Fe or metallic elements such as Al or Sn [54,55,56]. Another method of electrode modification is the deposition of manganese oxides on porous and high-surface-area materials with an electronically conducting structure. Carbon nanofoams, nanographite and templated mesoporous carbon are especially used for this process [57].

In this work, lignin was activated using selected ionic liquids, which was intended to lead to an increase in the activity of the biopolymer and secondly to facilitate the ability to bind activated lignin to manganese dioxide. An investigation was also made of the potential use of such a functional system as an innovative material in electrochemical applications. Functional hybrid materials combining MnO_2_ with lignin activated by ILs have not been previously described in the literature. In this work their physicochemical and morphological characteristics are determined, and potential uses in electrochemistry are identified.

## 2. Results and Discussion

### 2.1. Activation of Kraft Lignin in Ionic Liquids

#### 2.1.1. Fourier Transform Infrared Spectroscopy (FTIR) Analysis

**Figure 1:**
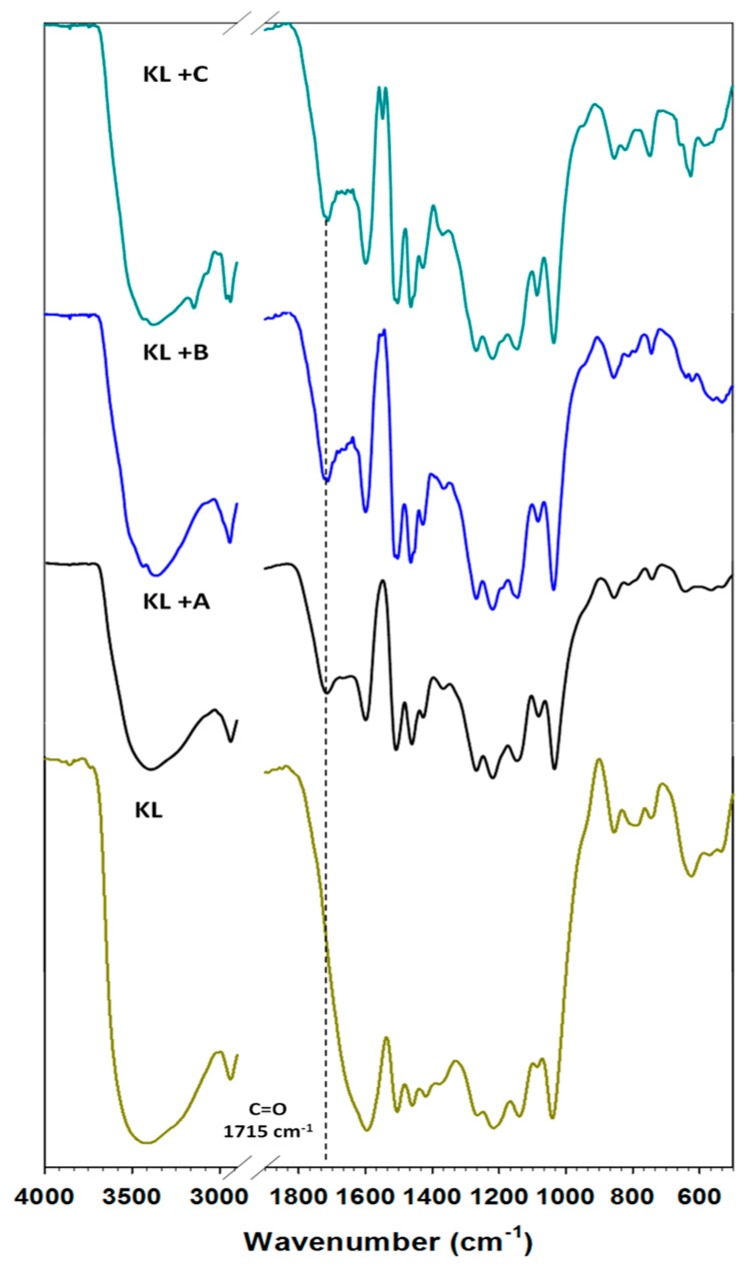
FTIR spectra of kraft lignin (KL) and activated lignin in selected ionic liquids: [C_4_im][HSO_4_] (KL+A), [Et_3_NH][HSO_4_] (KL+B) and [C_4_C_1_im][MeSO_3_] (KL+C). Dashed line: band derived from the carbonyl group.

The FTIR spectra of the kraft lignin used is presented in Figure 1, and the association of wavenumbers to functional groups is given in Table 1.

In the analyzed medium range of infrared spectroscopy for lignin, there occurred the following bands assigned to characteristic functional groups. A band for hydroxyl groups appeared in the range 3420–3390 cm⁻^1^. Stretching vibrations of C–H bonds occurred in the wavenumber range 2950–2800 cm⁻^1^, but deformation vibrations of the same bond appeared at 1450 cm⁻^1^. In the range 1600–1400 cm⁻^1^ bands attributed to aromatic units of the biopolymer were recorded. The most important band occurred at 1715 cm⁻^1^, attributed to unconjugated carbonyl groups. For unmodified lignin this signal was shifted and covered by the first characteristic signal from the aromatic ring (1600 cm⁻^1^). This biopolymer was used to investigate the activation process in selected ionic liquids. This method of modification was chosen as an eco-friendly and effective way to increase interaction with oxide in the prepared hybrid materials, and to develop their application potential. FTIR analysis was also performed for all of the activated lignins; the results are again shown in Figure 1. These biopolymers have the same characteristic bands as pure kraft lignin; however, the carbonyl signal is separated and better visible. For instance, activation in [Et_3_NH][HSO_4_] led to the most intensive signal. Therefore this ionic liquid was identified as the most effective activation agent of lignin, as is also confirmed by literature reports [27,39]. Furthermore, the FTIR spectra of the activated lignins contained more intensive bands attributed to C–H bonds at 2850 cm⁻^1^, which may demonstrate the partial depolymerization of aliphatic chains in the biopolymer. Corresponding results were observed in [39]. The main goal of that research was the oxidative depolymerization of lignin to low-weight organic compounds, but residual lignin was also observed, with the same changes as in the present work. From an economic point of view the activation of lignin at 60 °C is sufficient to obtain good-quality products for potential use in the preparation of functional hybrid materials.

#### 2.1.2. Elemental Analysis

Table 2 shows the elemental contents of nitrogen, carbon, hydrogen and sulfur in kraft lignin and activated biopolymer in selected ionic liquids. As expected, during analysis only contents of C, H and S were determined. The low value registered for nitrogen was attributed to impurities in the ionic liquids. The other elements examined were at the same level for kraft lignin and for the activated forms. These results confirm the success of the activation process in ionic liquids, with good cleaning of residual ILs.

#### 2.1.3. Morphology of Activated Lignin

**Figure 2:**
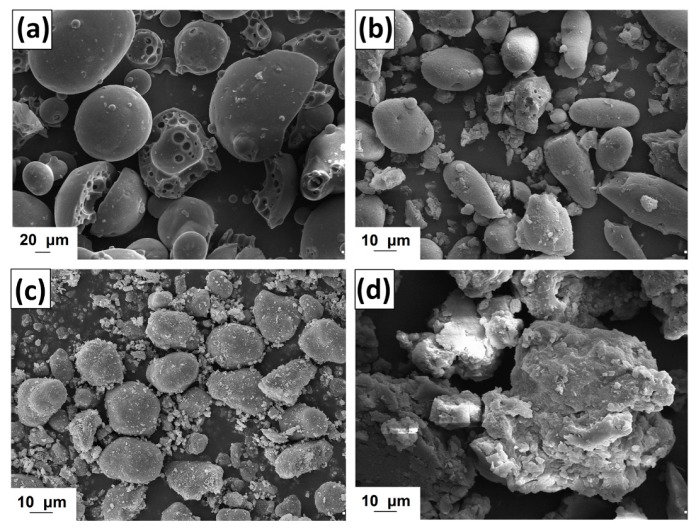
Scanning electron microscopy (SEM) images of lignin (**a**) and lignins activated by [C_4_im][HSO_4_] (**b**), [Et_3_NH][HSO_4_] (**c**) and [C_4_C_1_im][MeSO_3_] (**d**).

The morphology of kraft lignin and the activated products was evaluated based on SEM images, shown in Figure 2. The pure biopolymer has large (>20 μm) spherical particles with several circular holes located on their surface. The activation of lignin in selected ionic liquids promoted the depolymerization of biopolymer particles. In the first step the ionic solvent was able to dissolve lignin, which may take place by the partial depolymerization of aliphatic chains, as is confirmed by the FTIR results (see Figure 1). When lignin is isolated from the mixture, its particles tend to aggregate into larger structures, represented in Figure 2b–d. The final particles in activated lignins were smaller than in the pure kraft product. The biopolymer obtained in [C_4_im][HSO_4_] has particles with a shape tending to spherical, with partially developed surface microstructure. The smallest particles were observed for biopolymer activated in [Et_3_NH][HSO_4_], which has irregular shape and tends to aggregation. Lignin activated in [C_4_C_1_im][MeSO_3_] has larger, more irregular particles than previously discussed, although the surface area of this product is greater.

#### 2.1.4. Electrokinetic Properties

**Figure 3:**
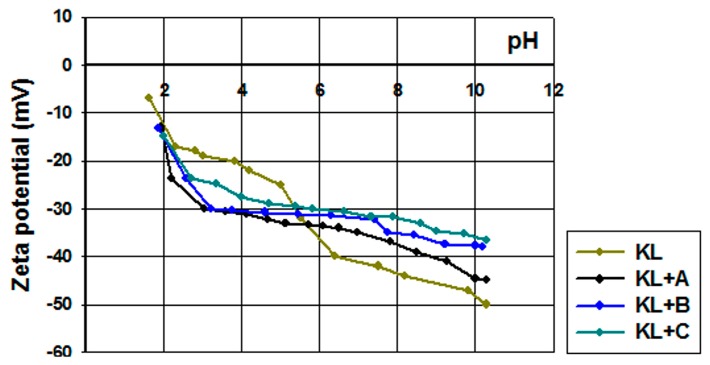
Zeta potential vs. pH for kraft lignin and activated lignins in selected ionic liquids: [C_4_im][HSO_4_] (KL+A), [Et_3_NH][HSO_4_] (KL+B) and [C_4_C_1_im][MeSO_3_] (KL+C).

For full characterization of surface functional groups, and to judge the potential of the obtained lignins for applications in electrochemistry, zeta potentials were determined in a pH range of 2–10 (Figure 3).

**Figure 4:**
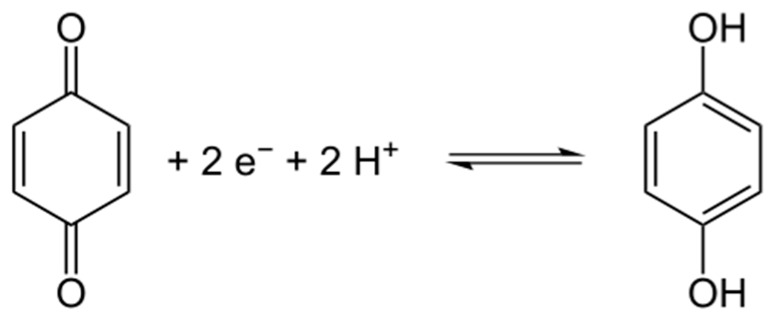
Schematic reduction of 1,4-benzoquinone.

Pure kraft lignin had negative surface charge over the measured pH range. For this reason, the biopolymer has a wide range of functional groups that can dissociate, for example hydroxyl groups, carboxyl groups and the very important carbonyl groups [62,63]. With decreasing pH of solution the total quantities of H^+^ and OH^−^ ions changed, having an effect on the biopolymer surface. According to the mechanism proposed by Dong et al. [63], the carbonyl groups dissociate and form a quinone-hydroquinone system. This is very important in electrochemistry, because this formation can transfer protons and electrons in electrochemical materials [64]. Kraft lignin attains electrokinetic stability above pH = 5, where the zeta potential is lower than −30 mV. In general the activated lignin surface also had negative charge in the measured pH range. It is for this reason that all of them have many functional groups that tend to dissociate in the solution used. Two of them reach electrokinetic stability earlier than kraft lignin, but on the other hand the biopolymer activated in [C_1_C_4_im][MeSO_3_] had less favorable properties. These values of zeta potential proved that the activation process of lignin with selected ionic liquids increases the quantity of dissociative functional groups. Potentially this procedure increases the quantity of carbonyl groups by oxidation and depolymerization of biopolymer. Moreover, based on the simple example of 1,4-benzoquinone, it is possible to transfer protons and electrons by reduction in a low pH range (see Figure 4). This mechanism of electron transfer was proposed by Milczarek and Inganas [64]. They suggest that lignin fragments like 1,2-benzoquinone could be reversibly oxidized and reduced. All of this evidence confirmed that the activation performed in ionic liquids was effective and improved the electrokinetic properties of the biopolymer.

#### 2.1.5. Thermal Analysis

**Figure 5:**
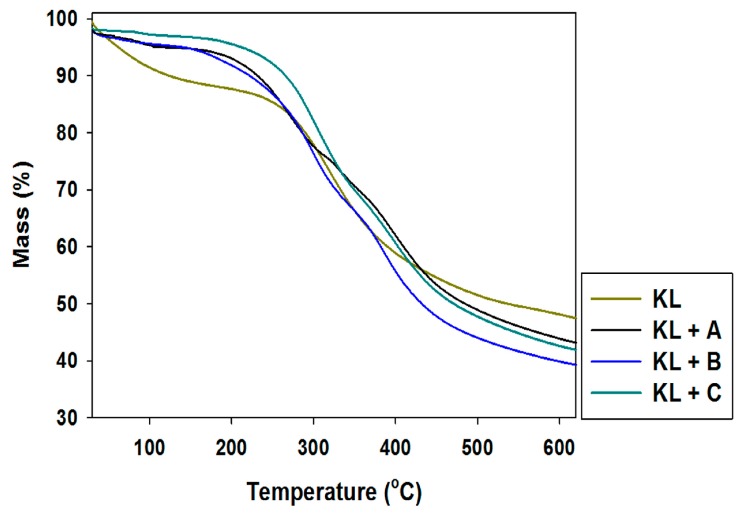
Thermogravimetric curves of kraft lignin (KL) and activated lignins by selected ionic liquids: [C_4_im][HSO_4_] (KL+A), [Et_3_NH][HSO_4_] (KL+B) and [C_4_C_1_im][MeSO_3_] (KL+C).

**Figure 6:**
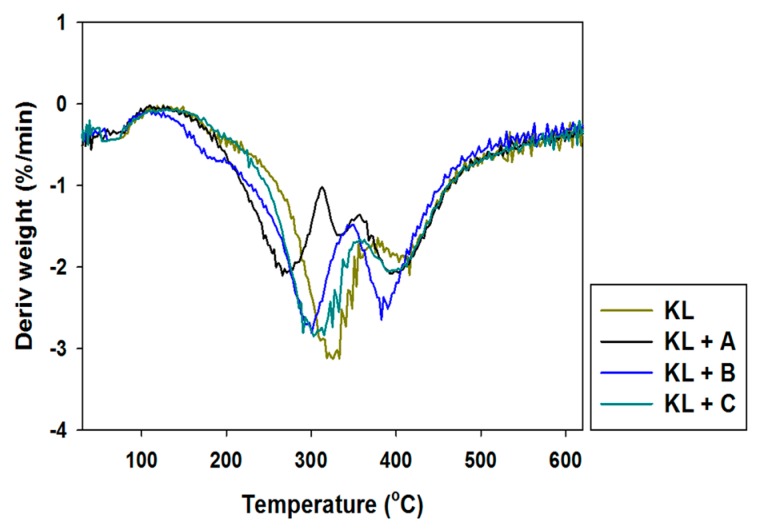
Differential thermogravimetry (DTG) curves of kraft lignin and activated lignins by selected ionic liquids.

To enable potential applications of the activated lignins to be proposed, their thermal stability was also determined. The obtained TGA curves are presented in Figure 5, and DTG curves in Figure 6.

Pure kraft lignin has two thermal degradation steps in the examined temperature range. The first step was recorded near 100 °C, associated with the removal of physically bound water. The next mass loss was observed above 300 °C, where some of the aliphatic chains were decomposed. In the investigated temperature range kraft lignin exhibited nearly 50% mass loss. Unfortunately, the activation of lignin in selected ionic liquids had no effect on the thermal stability of the biopolymer. The same conclusions were reached based on the DTG curves, where thermal changes in the biopolymer could be observed.

#### 2.1.6. Porous Structure Properties

Kraft lignin and biopolymer activated in ionic liquids underwent porous structure analysis (Table 3), involving the determination of the mean size (*S_p_*) and total volume of pores (*V_p_*) as well as BET surface area (*A_BET_*). The pure lignin has a low surface area of 1 m^2^/g. After activation of the biopolymer in selected ionic liquids there was observed a slight increase in this parameter, to 14–18 m^2^/g. With increasing value of the *A_BET_* parameter, the pore size decreases from 12.1 nm (KL) to 3.8–3.6 nm. All of the examined lignins have the same total pore volume, equal to 0.01 cm^3^/g. The results confirm that the proposed activation of lignin takes place by partial depolymerization, which is also observed on the SEM images (Figure 2). This occurs as a result of the breaking of bonds (especially β*-O-*4 ether linkages) in lignin under the influence of ionic liquid.

#### 2.1.7. Regeneration of Ionic Liquids

**Figure 7:**
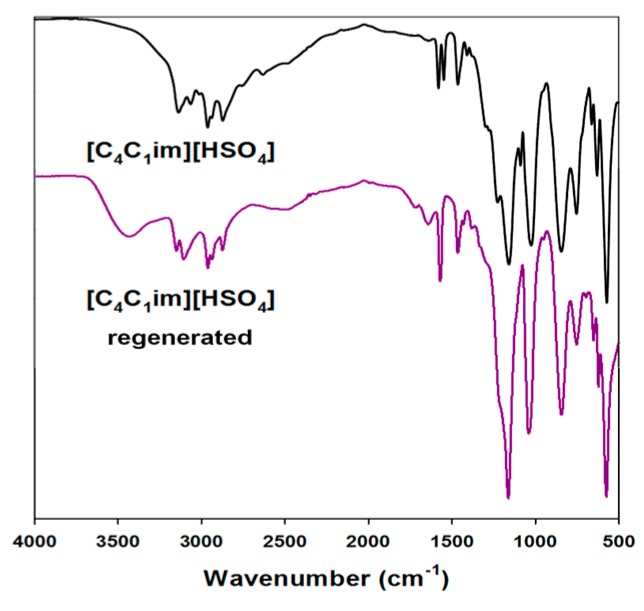
Attenuated total reflection (ATR) spectra of used and regenerated ionic liquids.

A proposed ionic liquid regeneration procedure was developed on the basis of literature reports [44]. This method was performed for all of the ILs used, but only the results for [C_4_im][HSO_4_] are shown here (Figure 7).

The main difference between the spectra of the pure and regenerated ILs was the presence of a band attributed to hydroxyl groups. This is probably due to residual lignin or possibly water which was not fully reduced from [C_4_im][HSO_4_]. Even after a few days of heating at a temperature of 60 °C, this band still exists. This shows that impurity is combined not only by physical interactions, but also on a chemical level. Moreover, in the infrared range from 1600 to 1400 cm⁻^1^ there were observed weak bands associated with aromatic rings, specific to the biopolymer used. The remaining bands on the ATR spectra correspond between pure and regenerated ILs. The regeneration process was performed for all of the ionic liquids used, and the results indicated the same level of efficiency (data not shown).

### 2.2. Functional MnO_2_-Activated Lignin Hybrid Materials

#### 2.2.1. FTIR Analysis

**Figure 8:**
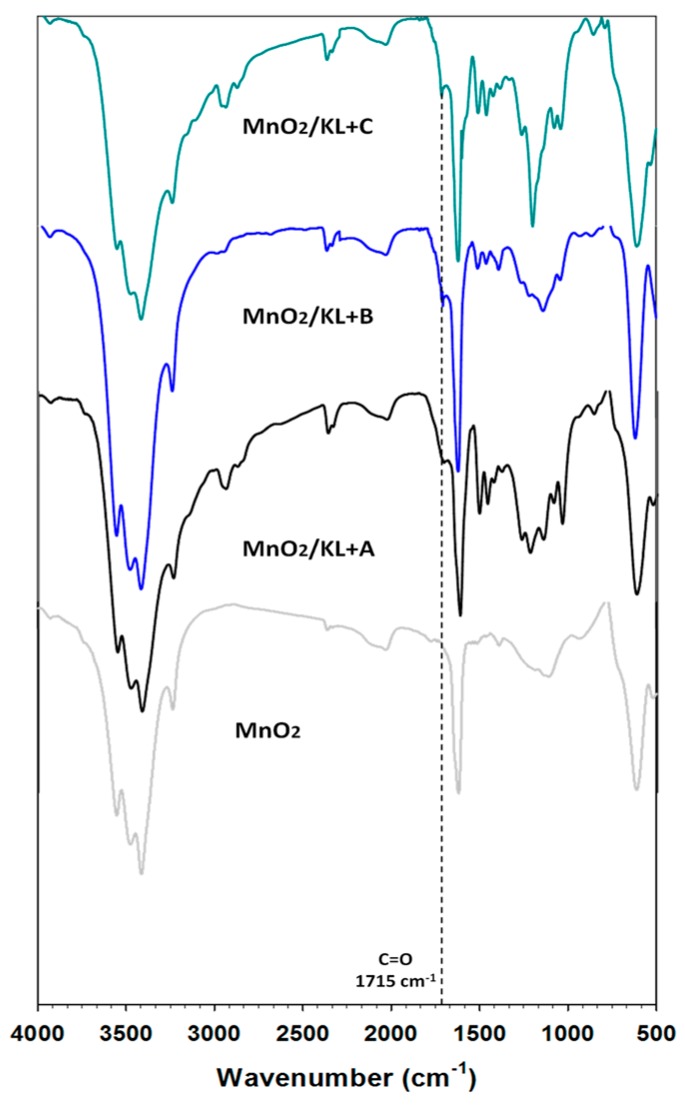
FTIR spectra of manganese oxide and MnO_2_/lignin hybrid materials based on activated biopolymer with using selected ionic liquids: [C_4_im][HSO_4_] (MnO_2_/KL+A), [Et_3_NH][HSO_4_] (MnO_2_/KL+B) and [C_4_C_1_im][MeSO_3_] (MnO_2_/KL+C).

On the spectrum of manganese oxide (Figure 8) a strong broad band was observed at wavenumbers 3600–3400 cm⁻^1^, attributed to stretching vibrations of hydroxyl groups located on the oxide surface or physically adsorbed water. The presence of these functional groups is also registered as a single strong signal at 1630 cm⁻^1^ [65,66]. Next in the spectrum absorption bands were observed at around 598 cm⁻^1^ and 527 cm^−1^, which is characteristic for vibrations of the Mn-O linkage [65,66].

For the prepared hybrid materials the presence of activated lignin was also observed, based on new weak bands and those characteristic for the biopolymer. All spectra contained a wide band attributed to hydroxyl groups at about 3500 cm⁻^1^ (represented on all spectra of lignin and manganese oxide), and stretching vibrations characteristic for C–H bonds (2900 cm⁻^1^), which are characteristic for aliphatic and aromatic rings. The very important band attributed to carbonyl groups (1715 cm⁻^1^) was decreased. Probably these groups react with hydroxyl groups on the oxide surface with the creation of hydrogen bonds. However for the hybrid material based on lignin activated in [C_4_C_1_im][MeSO_3_], the carbonyl group band still exists on the spectrum. Also present were three characteristic bands attributed to aromatic rings (1600–1400 cm⁻^1^). At wavenumber 1155 cm⁻^1^ there were observed deformation vibrations of O=S=O groups. FTIR analysis confirmed the effective preparation of novel hybrids, where both precursors react with the creation of hydrogen bonds between oxygen from carbonyl groups (lignin) and hydroxyl groups on the oxide surface.

#### 2.2.2. Elemental Analysis

The results of determination of the contents of basic elements are presented in Table 4. The manganese oxide shows low contents of carbon and hydrogen, as expected. The prepared MnO_2_/lignin hybrid materials have higher contents of carbon, hydrogen and sulfur. The obtained materials were prepared by mixing manganese oxide with activated lignins in an equal mass ratio. This ratio was reflected in the analysis; compared with Table 2 the contents of the basic elements decreased by half. As noted previously in Section 2.1.2, the low values of the elements nitrogen and sulfur came from residual ionic liquid. In summary, these results confirm the successful preparation of functional hybrid materials.

#### 2.2.3. Morphology of MnO_2_/Lignin Hybrids

**Figure 9:**
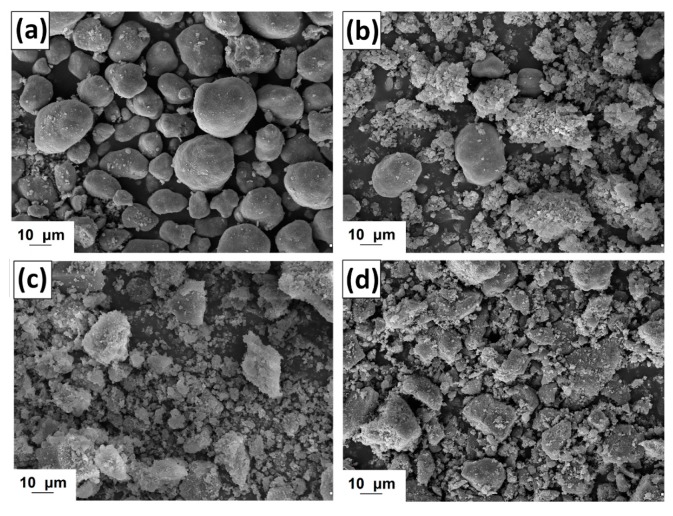
SEM images of manganese oxide (**a**) and MnO_2_/lignin hybrid materials based on biopolymer activated by using [C_4_im][HSO_4_] (**b**), [Et_3_NH][HSO_4_] (**c**) and [C_4_C_1_im][MeSO_3_] (**d**).

SEM microphotographs for manganese oxide and hybrid materials with lignin activated in [C_4_im][HSO_4_] and [C_4_C_1_im][MeSO_3_] are shown in Figure 9.

The oxide used has large irregular particles with shape tending to spherical. However the surface area of this material is large, as is confirmed by the measured of parameters of porous structure (see Table 5). On the other hand the prepared MnO_2_/lignin hybrid materials have non-homogeneous particle size distributions. The SEM images show primary, irregular particles with a tendency to form aggregates. These particles potentially originate from the activated lignin. The larger ones were similar to MnO_2_ particles. The mechanical process of preparation of the hybrids had an influence on their morphology, because some of the particles were crushed, especially the lignin ones.

#### 2.2.4. Electrokinetic Properties

**Figure 10:**
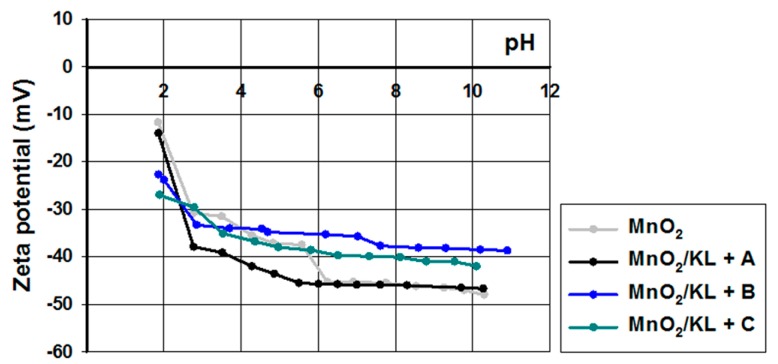
Zeta potential vs. pH for MnO_2_ and hybrids based on activated lignin.

The electrokinetic properties of the examined hybrids were determined based on the values of zeta potential; the obtained curves are shown in Figure 10. The zeta potential of manganese oxide was stable in almost the whole examined pH range. Already at pH = 3 it reached a zeta potential of below −30 mV, where kraft lignin and the activated lignins were unstable (see Figure 3).

On the other hand the prepared hybrids were equally stable as pure manganese oxide. Moreover, all of the examined samples reach equilibrium values of zeta potential above pH = 3. These results indicate the possibility of performing subsequent experiments regarding electrochemical applications of these materials.

#### 2.2.5. Thermal Stability

**Figure 11:**
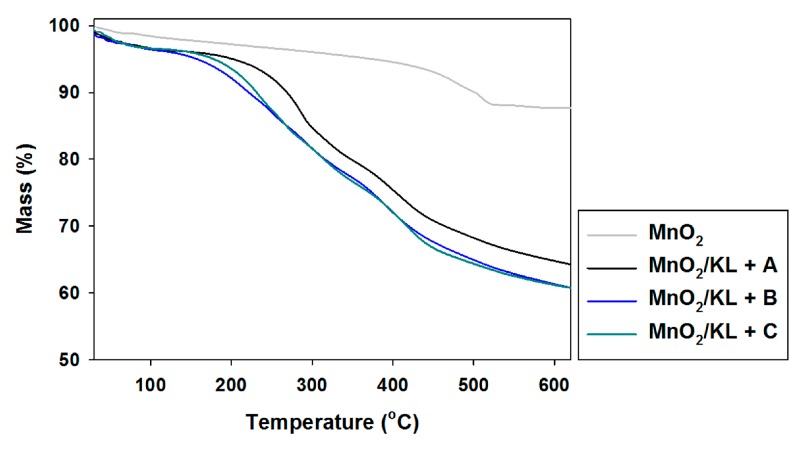
TGA curves of manganese oxide and hybrids obtained by combination of MnO_2_ and activated lignins using selected ionic liquids: [C_4_im][HSO_4_] (MnO_2_/KL+A), [Et_3_NH][HSO_4_] (MnO_2_/KL+B) and [C_4_C_1_im][MeSO_3_] (MnO_2_/KL+C).

**Figure 12:**
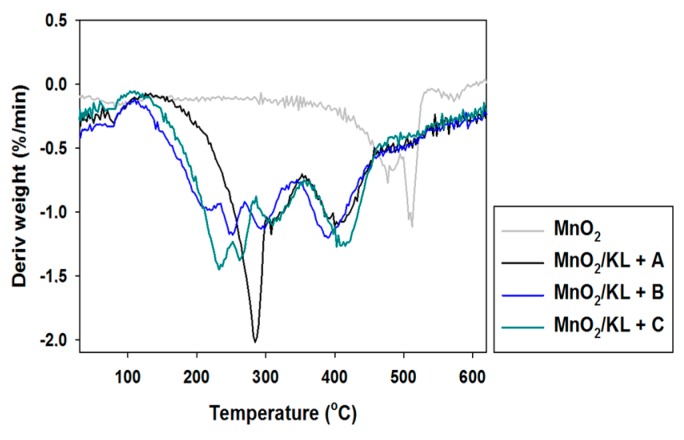
DTG curves of manganese oxide and hybrid materials.

The thermogram for manganese oxide (Figure 11) shows a gentle decrease in mass with increasing temperature, reaching equilibrium at 550 °C with 10% mass loss. This is confirmed by DTG analysis, where only one drop in mass was recorded above 500 °C. The addition of activated lignin causes deterioration in the hybrid materials’ thermal stability (see Figure 11 and Figure 12).

In the examined temperature range there was observed a gentle decrease (almost 40%) in the mass of the products. However the best additive was lignin activated in [C_4_C_1_im][MeSO_3_], which caused thermal degradation to begin near 300 °C, where in the remaining cases it began at 220 °C. Based on the DTG curves (see Figure 12) of the prepared hybrid materials there were observed two or three falls in mass in the investigated temperature range. This situation corresponds to the DTG curves shown in Figure 6, and is characteristic for the presence of the activated biopolymer in the prepared hybrids. In comparison with the TGA curves obtained for activated lignins (Figure 5), combination with manganese dioxide minimally increases the thermal stability of the final materials. Moreover, these results were predictable based on TGA and DTG curves determined for pure lignins and manganese oxide. On the other hand, these materials have poorer thermal stability than the SiO_2_/lignin hybrids well known from the literature [21,22,23].

#### 2.2.6. Porous Structure Properties

Manganese oxide has a relatively large BET surface area (*A_BET_*) of 98 m^2^/g, with small pores. The results for the oxide and the prepared hybrid materials are presented in Table 5. Addition of activated lignin reduced the value of the *A_BET_* parameter, but without influence on the pore size and volume. The observed decrease in the determined parameters reflected the presence of the biopolymer in the prepared hybrid materials. For example, the BET surface area of lignin activated in [C_4_im][HSO_4_] is equal to 18 m^2^/g, but in the prepared hybrid it increased to 50 m^2^/g. The lowest values were obtained for material based on MnO_2_ and lignin activated in [C_4_C_1_im][MeSO_3_] (34 m^2^/g). The others have BET surface areas near 50 m^2^/g. Moreover, the addition of activated biopolymer did not affect the pore volume and size of the obtained material, in comparison with the pure oxide. To sum up this surface analysis, it was easy to predict the direction of changes in the determined parameters. However, the results do not exclude the application of these materials for electrochemistry, since more important in that regard are the functional groups located on the surface of the material, which are able to transfer protons and electrons. These properties were determined previously in Section 2.1.4.

### 2.3. Electrochemistry Data

#### 2.3.1. Impedance Spectra

**Figure 13:**
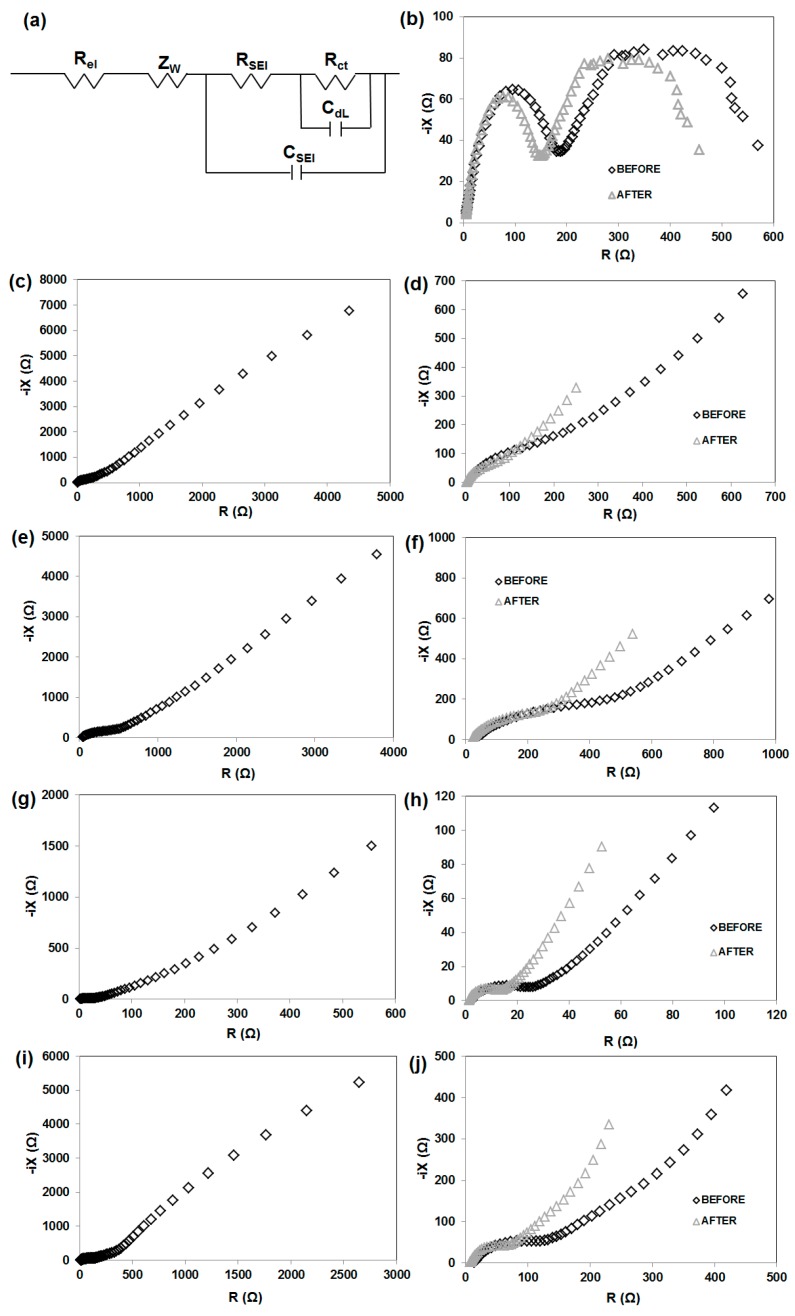
Equivalent electrical circuit (**a**), and EIS measurements of MnO_2_|Li (**b**), KL|Li (**c**) MnO_2_/KL+A|Li (**e**), MnO_2_/KL+B|Li (**g**) MnO_2_/KL+C|Li (**i**); plots magnification before and after charging-discharging for KL|Li (**d**) MnO_2_/KL+A|Li (**f**), MnO_2_/KL+B|Li (**h**) and MnO_2_/KL+C|Li (**j**) systems.

To confirm the cycle stability of MnO_2_|Li, KL|Li, MnO_2_/KL+A|Li, MnO_2_/KL+B|Li and MnO_2_/KL+C|Li, the electrochemical impedance spectroscopy (EIS) measurements are conducted and the results are shown in Figure 13.

The Nyquist plots (before charging-discharging) for all four electrodes are similar (excluding MnO_2_—Figure 13b), and show one semicircle in the high–middle-frequency region and an inclined line in the low-frequency region. The diameter of these circles is dependent on the interface contact and charge transfer resistance [49]. The MnO_2_/KL+C electrode has a clearly smaller diameter of the semicircle compared with MnO_2_/KL+A. This difference supports the finding that lignin (KL) can facilitate electron and Li-ion transfer and improve electrical conductivity, thus resulting in significant improvement in the electrochemical performance of MnO_2_ [49]. Moreover the impedance range for pure lignin excluded that electrode from further electrochemical studies (Figure 13c).

**Figure 14:**
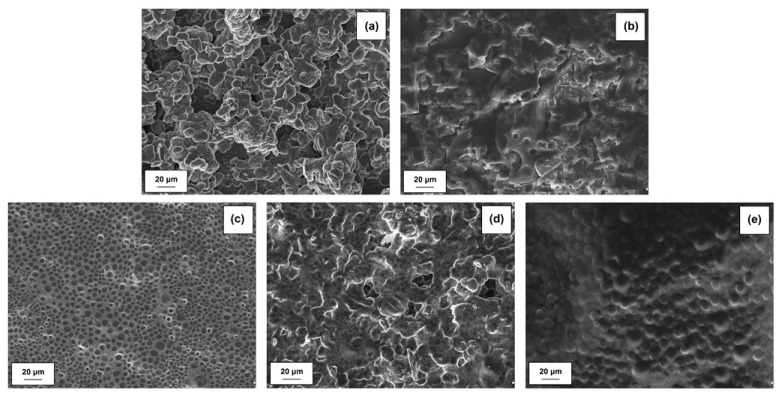
SEM images of electrodes: MnO_2_ (**a**), KL (**b**), MnO_2_/KL+A (**c**), MnO_2_/KL+B (**d**) and MnO_2_/KL+C (**e**).

The EIS results also show the impedance of the anode interface. Nyquist plots for MnO_2_|Li cells without activated lignin composition is shown in Figure 13b. Typically, spectra taken immediately after cell assembly consist of two parts in the high-frequency region. On the other hand, the impedance plots consisting of two semicircles may also be attributed to the anode/electrolyte interface (the high-frequency semicircle) and to the cathode/electrolyte interface (the low-frequency semicircle). Confirmation is seen on SEM images (see Figure 14). It is clearly observed that irregularly shaped pores are randomly located on the surface of the electrode spheres (Figure 14a,b,d). A different structure is observed for MnO_2_/KL+A and MnO_2_/KL+C (Figure 14c,e )—the change in structure is caused by the ILs used.

Low potential anodes (e.g., lithium or lithiated graphite) exhibit a tendency to spontaneously react with electrolytes. In case of metallic lithium as well as lithiated graphite it is necessary to include a protective coating, as lithium may grow dendrite crystals on its surface. As a result, it is expected that the electrolyte used for Li-ion batteries will form an SEI which will protect the anode [67]. A similar phenomenon was observed for the presented anode materials. A solid electrolyte interface can be formed after several cycles e.g., on the MnO_2_/KL+B anode. In contrast the SEI film on a graphite anode is formed during the initial cycle [68,69]. Impedance spectroscopy may be used in order to investigate the passivation of electrodes in a Li-ion battery (Figure 13b,d,f,h,j). Electrochemical impedance spectra (EIS) of electrode|electrolyte|Li were measured before and after 20 cycles (at a current of 50 mA·g⁻^1^). Two electrode cells were used to record the impedance spectra with the use of an Li counter. The impedance of the interface of lithium was included in the EIS results. It can be observed that the spectra obtained directly following the cell assembly included a semicircle and a linear part. The semicircle may be associated with e.g., polarization resistance or SEI layer formation, whereas the linear part may be attributed to the diffusion of Li ions (in the electrolyte, SEI or the solid electrode). The high diffusion resistance of lithium may be a rate-determining factor in the anode material. The semicircle and long line were present in the impedance spectrum obtained for 1M LiPF_6_ (in EC:DMC, 1:1) during charge/discharge. However, after charging/discharging one, much smaller semicircle was present in the spectrum.

The resistance of the SEI layer, *R*_SEI_, formed before and after charging/discharging on anode were listed in Table 6. All value are much smaller after galvanostatic charging-discharging. A possible explanation is that at a higher temperature the thickness of the SEI layer formed was greater.

The charge transfer resistance, *R*_ct_, of the MnO_2_/KL+B|Li^+^ anode was ca. 31 Ω, decreasing to ca. 18 Ω after charging/discharging (see Table 6). The charge transfer resistance of MnO_2_/KL+B electrode is 18 Ω, which is slightly lower than that of MnO_2_/KL+A electrode (205 Ω), and MnO_2_/KL+C (80 Ω) confirming that the electronic conductivity of MnO_2_/KL+B electrode is better than that of another two electrodes. Hence, this is one of the reasons that the specific capacity of MnO_2_/KL+B is higher.

The diffusion of electro-active species may be attributed to the line visible in the low-frequency region. In general, the Warburg model based on a symmetrical constant phase element is applied by all software responsible for the deconvolution of the EIS curve. Therefore the approximation of the diffusion process is based on the Z_w_ (the Warburg element). In case of the linear parts, the slopes were not at 45° in contrast to the preductions of the Warburg model. Furthermore, an increase of the diffusion impedance with the increase of temperature was observed in case of the MnO_2_/activated lignin|Li^+^ anode, however the explanation of this phenomenon is difficult. On the other hand, a considerable decrease of the diffusion impedance with the increase of temperature was observed in case of the MnO_2_/activated lignin anode.

Moreover, it is considered that the semicircle in the low frequency region may be attributed to ohmic resistance, which corresponds to the electrolyte R_el_ in the equivalent circuit. The resistance associated with the charge transfer occurring on the electrode/electrolyte interface, which is related to a parallel circuit element, may correspond to the semicircle in the medium frequency region. This corresponds to the capacitance of the double layer C_dl_ as well as the resistance of charge transfer *R*_ct_ in the equivalent circuit. The linear part, associated with the diffusion of Li ions in the electrode, may correspond to Z_w_ (the Warburg element) in the equivalent circuit [70]. Further explanations may be found in corresponding literature reports [71,72,73]. The obtained data was analysed by fitting equivalent circuit [70].

The transition metal oxides (TMOs) are another group of studied anode materials, which are mostly characterized by a specific mechanism of lithium storage in comparison to the two previous types of metal oxides. The resulting forward displacement redox reaction involving MO and Li^+^ is favourable in terms of thermodynamics. Usually multiple-electron transfers per a single metal atom are required for the conversion of metal oxides to their metallic state, which results in a high theoretical storage capacity of lithium [74,75].

However, it seems that the reversed reaction of extracting Li^+^ from Li_2_O cannot be achieved thermodynamically under normal conditions. The formation of metal nanoparticles (M) upon first discharge may facilitate the reversible formation and decomposition of the Li_2_O matrix, and the use of nanostructured materials possessing large surface area, high surface energy and enhanced electrochemical reactivity can also facilitate the backward reaction. The nanoscale facilitates the conversion reaction and improves its reversibility. Aside from the abovementioned potential thermodynamic and kinetic limitations associated with lithium diffusion, it has also been established that the reorganization of structure and variation of volume resulting from the charge/discharge may also lead to the cracking of active materials and rapid decrease of their capacity. It may possible to overcome such restrictions of TMO-based materials by preparation of specific nanostructures which would sustain structural disintegration and facilitate reactions associated with lithium storage.

#### 2.3.2. Cyclic Voltammetry

**Figure 15:**
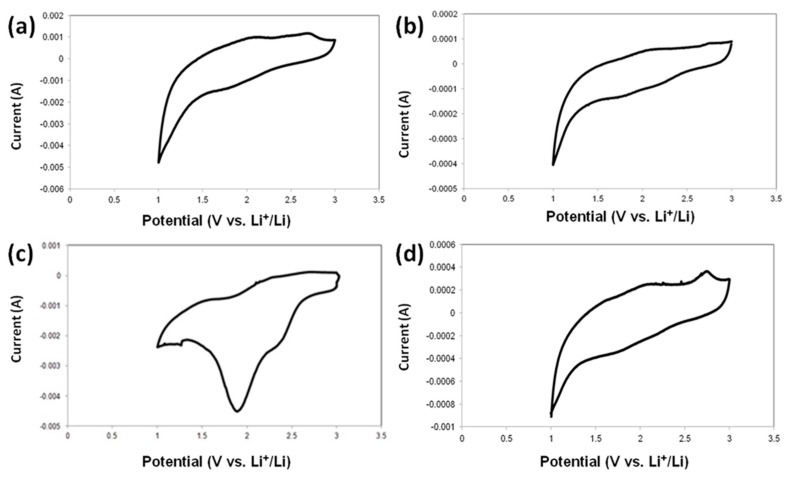
Cyclic voltammetry curves of MnO_2_|Li (**a**), MnO_2_/KL+A|Li (**b**), MnO_2_/KL+B|Li (**c**) and MnO_2_/KL+C|Li (**d**).

Figure 15 shows the cyclic voltammograms of MnO_2_, MnO_2_/KL+A, MnO_2_/KL+B and MnO_2_/KL+C electrodes in the potential range from 1 to 3 V (versus Li/Li^+^) at a scan rate of 0.2 mV s^−1^ for the first cycle. Notably, the redox curves of the first two samples intersect at a low potential.

It can be seen that the shapes of the CV curves for the four samples are similar, indicating that the reduction and oxidation process of the prepared MnO_2_/KL+IL samples are consistent (with the exception of Figure 15c). As seen in Figure 15b–d, the three peaks at 2.35 V; 1.90 V and 1.01 V in the cathodic scan, which may arise from the formation of a solid electrolyte interface (SEI) layer and can be assigned to the electrochemical reduction of MnO_2_ with Li, respectively [76]. The shifts may be associated with the presence of activated lignin. The oxidation peaks centered at 1.23 V in the anodic scan are characteristic for the oxidation reaction. Significant flattening of the peaks in oxidation and reduction can indicate that the MnO_2_/KL+A and MnO_2_/KL+C have an immense polarization effect.

Although acceptable performances were not yet reached for the majority of applications due to low output voltage, this first attempt presents some advantages of MnO_2_ and activated lignin that react with Li through two-step processes, separated by a potential. Since each ΔV depends on the potential values of each respective process, they can be modified using chemical methods (e.g., by changing the nature of active species, substitution of functional groups, or addition of a modifier), in order to either separate or merge the two redox processes. Of course, this requires further analysis of quinone groups and their potential impact on electrochemical processes [77].

Other studies report the use of polymers [78,79,80] and radical polymers [81,82,83] or organic radicals as possible electro-active materials in Li-ion batteries. Working with such systems is associated with many issues therefore researchers are looking for new solutions. Authors demonstrated the principle by grafting a quinine derivative of calix[4]arene onto the surface of two different substrates: (a) nanosized silica particles and (b) selected carbon black samples. Although this approach has several limitations, it can in principle be enhanced by the use of appropriate novel solutions. The energy density lost as a result of grafting on inactive substrate may be efficiently restored by the use of organic compounds with lower molecular mass or substrates which exhibit very high surface area. In addition, high loading of redox active molecules may be alternatively achieved by construction of electrode composites similar to those proposed recently in Li–S batteries [84]. Recent literature reports confirm that strong interactions between organic compounds and the surface of carbon may be achieved during their electrochemical treatment [85,86]. As a result, effective grafting of specific compounds onto a substrate may potentially be obtained by employing electrochemically assisted covalent modification of substrates.

The authors observed more pronounced peaks of oxidation and reduction processes only in Figure 15c. This property has shifted to a higher stiffness of this electrode during the charging/discharging process.

#### 2.3.3. Charging/Discharging

**Figure 16:**
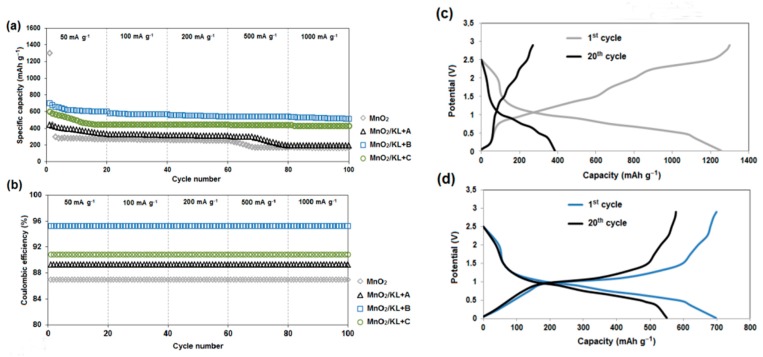
Rate capability of the systems (**a**); coulombic efficiency (**b**); charge/discharge curve of pure MnO_2_ (**c**) and charge/discharge curve of MnO_2_/KL+B (**d**).

Figure 16a displays the rate performance of MnO_2_, MnO_2_/KL+A, MnO_2_/KL+B and MnO_2_/KL+C electrodes at 50 mA·g⁻^1^, 100 mA·g⁻^1^, 200 mA·g⁻^1^, 500 mA·g⁻^1^ and 1000 mA·g⁻^1^ for 20 cycles each. The results show that the specific capacity decreases with increasing current density, since high current density causes a low rate of the Li-ion diffusion [87].

The MnO_2_/KL+B delivers specific capacities of 760, 605 and 587 mAh·g⁻^1^ at current densities of 50, 100 and 200 mA·g⁻^1^, respectively. Even at a current density of 1000 mA·g⁻^1^, the MnO_2_/KL+B electrode still maintains a specific capacity as high as 520 mAh·g⁻^1^. Remarkably, all of these values are much higher than for MnO_2_/KL+A, MnO_2_/KL+C and MnO_2_, suggesting that the rate capability of the MnO_2_/KL+B electrode is superior in comparison with the other three electrodes. The excellent electrochemical performance of the MnO_2_/KL+B composite may be attributed to the unique porous structure, which provides many large electrochemical sites and a short pathway for Li-ion diffusion, and limits the volume change, as well as the synergistic effect of activated lignin (with ionic liquid–triethylammonium hydrogen sulfate) and MnO_2_ [51,88,89].

Numerous studies have been carried out on the characterization of the SEI (solid electrolyte interphase) on the negative electrode in Li-ion batteries. The chemical composition at a detailed level is dependent on the type of salt present in the electrolyte, temperature, type and surface chemistry of the anode, among other parameters [90].

Firstly, the activated lignin coating most likely prevents the direct exposure of MnO_2_ to the electrolyte, and thus maintains the structural and interfacial stabilization. Secondly, the activated lignin coating favors formation of a stable SEI layer owing to the improved surface chemistry state of MnO_2_. Thirdly, the activated lignin can accommodate the large volume change of MnO_2_ and thus improve the electrochemical activity. Fourthly, the activated lignin provides abundant electroactive sites for Li-ion storage. In addition, the components in the MnO_2_/activated lignin have a synergistic effect on the cycling stability. It should be noted that all of the electrodes (MnO_2_/activated lignin) had stable capacities and no significant drop in stability was observed as in the case of pure MnO_2_.

The charging/discharging profiles for the electrodes of MnO_2_ and MnO_2_/KL+B at a current of 50 mA·g⁻^1^ are shown in Figure 16c,d. It can be clearly observed that the MnO_2_/KL+B electrode has a much better capacity performance and cycling stability. After 20 discharging and charging cycles, it delivers a discharge capacity of 750 mAh·g⁻^1^, whereas that of the MnO_2_ electrode is only 250 mAh·g⁻^1^. Moreover, after the initial cycles, the Coulombic efficiency (Figure 16b) can reach up to 98.5%, which further indicates that the MnO_2_/KL+B electrode has good reversibility. The lowest value is observed for pure MnO_2_ (Coulombic efficiency: 86%), which is due to significant decrease in capacitance during charging and discharging and poor electrode reversibility. For other electrodes, the Coulombic efficiency is similar: MnO_2_/KL+A = 89% and MnO_2_/KL+C = 91%, respectively.

There was one plateau at approx. 1.1 V in the charge process and one plateau at approx. 0.47 V in the discharge process (Figure 16c–d). The shape of the charge-discharge curves is typical for the conversion reactions of transition metal oxide anode materials. It has been found that the reaction leads to formation of nano metal clusters embedded in Li_2_O matrix, accompanied by enormous structural change [91,92].

The gradual increase of capacity in case of nanosize metal oxide anodes, may potentially result from a mechanism associated with interfacial storage of metal oxides nano domains due to the amorphization process which occurs during cycling [93,94,95]. Both the rate capability and cycle stability of the composite were similar in comparison to other reports regarding manganese dioxide anode materials [51,96,97,98].

Moreover the practical capacity provided by the transition metal oxide and phosphate anodes has reached its maximum intrinsic limit (140–170 mAh·g⁻^1^). In this respect, there is an increasing demand for the development of cathode and anode materials which have higher capacity [99,100]. Organic electrodes offer high theoretical capacity (300–800 mAh·g⁻^1^) due to possible low molecular weight structure and multiple electron redox reaction [101]. Their electrochemical properties could be finely tuned by structural modification. Although properties of organic compounds are desirable, critical issues such as dissolution of electrodes in liquid electrolytes, low electronic conductivity, irreversibility and low cyclability, should be addressed. Recently organic electrode materials are revisited by many research groups [101,102].

One of the main challenge in the development of organic electrode material is the capacity loss due to dissolution of the material in liquid electrolytes. Attempts have been made to tackle this problem by optimization of molecular structure, polymerization, using higher content of carbon, low solubility electrolyte etc. But one of them completely eliminates the problem of dissolution and gives higher capacity [78,80,103,104,105]. Therefore the fundamental solution to dissolution of electrode material is solid state battery. Solid state batteries are gathering increasing attention compared to the liquid electrolyte based battery due to their potential advantages over the latter [106,107]. In this regard, it is highly important to study the organic electrodes in conjunction with the electrolytes.

It has been reported that materials based on metal oxides exhibit increased lithium storage performance as a result of their efficient traits in terms of Li^+^ ion insertion and buffering of volume during the charge-discharge process [108,109]. Additionally, the hybrid core/shell structures exhibited improved electrochemical properties compared to those based on a single component as a result of higher surface areas and integral configurations [110,111].

In the table 7 are listed the new composite-electrode materials for comparison with the anode presented in this paper.

Moreover many efforts have been done in the investigation of both carbon and non-carbon materials for high performances and high capacity anode in LIBs. A short list must include: carbon nanotubes (1100 mAh·g⁻^1^) [112], carbon nanofibers (450 mAh·g⁻^1^) [113], porous carbon (800–1100 mAh·g⁻^1^) [114], SiO_x_-based anodes (1600 mAh·g⁻^1^) [115], germanium (1600 mAh·g⁻^1^) [116], tin (994 mAh·g⁻^1^) [117] and transition metal oxides (500–1000 mAh·g⁻^1^) [118,119,120]. Furthermore, metal sulphides, phosphides and nitrides [121,122,123] might be also considered for anodes purposes, in fact they possess specific capacity higher ca. 500 mAh·g⁻^1^. However, high volume expansion, poor electron transport, capacity fading, and low coulombic efficiency as well, are the main limitations that have to be overcame before they can be used as effective anodes. Compared to the literature (Table 7), the presented material, in particular its capacity, falls within the confines of other scientists.

## 3. Materials and Methods

### 3.1. Process of Kraft Lignin Activation in Ionic Liquids

**Figure 17:**
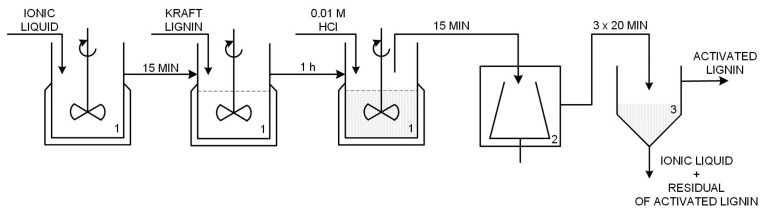
Technological process for the activation of lignin by the ionic liquids (1—reactor, 2—centrifuge tube, 3—gravity filter).

Kraft lignin with M_w_ of ~10,000 (Sigma-Aldrich, Hamburg, Germany) was used. This biopolymer was modified using ionic liquids: (*i*) 1-butylimidazolium hydrogen sulfate [C_4_im][HSO_4_] synthesized based on 1-butylimidazole (Sigma-Aldrich, Hamburg, Germany, assay > 98%) and sulfuric acid solution 98% (Chempur^®^, Piekary Slaskie, Poland, assay > 98%), based on the process described in [39]; (*ii*) triethylammonium hydrogen sulfate [Et_3_NH][HSO_4_] from triethylamine (Sigma-Aldrich, Hamburg, Germany, assay > 99%) and sulfuric acid, whose preparation was also reported in [39]; and (*iii*) 1-butyl-3-methylimidazolium methane sulfonate [C_4_C_1_im][MeSO_3_] (assay > 95%). Activation was carried out in an EasyMAX^TM^102 reactor (Mettler Toledo, Greifensee, Switzerland) as follows: 8 g of lignin was dissolved in 20 g of selected ionic liquid and stirred (400 rpm) for 60 min; after that time 40 mL of 0.01 M hydrochloric acid was added into a reaction flask using a syringe pump. After 15 min the whole mixture was transferred to a centrifuge tube and placed in a Centrifuge 5810R (Eppendorf AG, Hamburg, Germany) three times for 20 min. Each centrifugation was performed at 5 °C and 4000 rpm. In the next step the precipitate was separated by gravitational filtration and finally dried at room temperature. The resulting dried powder was sieved and its physicochemical properties were determined. A simplified diagram of the technological process of lignin activation in ionic liquids appears in Figure 17.

### 3.2. Regenreation of Ionic Liquids

**Figure 18:**
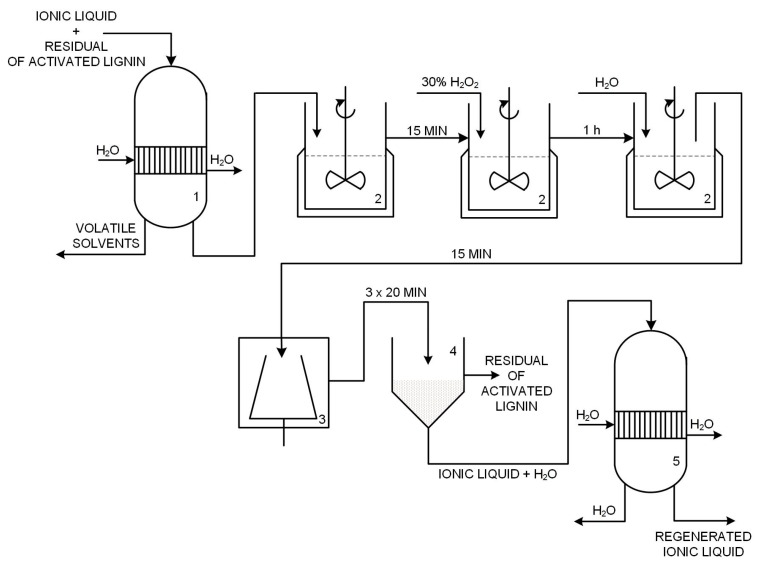
Technological scheme of regeneration of ionic liquids (1, 5—vacuum evaporator, 2—reactor, 3—centrifuge tube, 4—vacuum filter).

In the gravitational filtration process the reaction mixture was collected with an ionic liquid. This aqueous solution of IL was transferred to a rotary evaporator to remove volatile solvents. The operation was performed using a Rotavapor RII rotary evaporator with a Vacuum Controller V-850 vacuum pump (BÜCHI Labortechnik AG, Flawil, Switzerland). Water evaporation from ILs was carried out at 60 °C and 72 mbar. The water-free ionic liquid was re-introduced into the 100 mL glass EasyMax^TM^ 102 reactor and heated to 60 °C with constant stirring at 400 rpm. Then 5% by weight of a 30% H_2_O_2_ solution of IL + lignin was added, and the heating and stirring continued for 1 h. This addition of hydrogen peroxide corresponds to previous literature reports [44]. The oxidized biopolymer was precipitated with the addition of a double volume of water, calculated according to the initial weight of IL + lignin. Precipitation was carried out with continuous stirring for 15 min at 60 °C. The precipitated lignin was isolated from the mixture by 10 min of centrifugation at 5 °C and 4000 rpm. Finally, the oxidized biopolymer was filtered off under reduced pressure in a Sartorius AG filter kit (Goetzingen, Germany). The collected IL was separated from the water by means of a vacuum evaporator which maintained the temperature at 60 °C and lowered the pressure to 72 mbar. Figure 18 shows a simplified diagram of the regeneration of ionic liquids.

### 3.3. Preparation of MnO_2_/Lignin Hybrid Material

**Figure 19:**
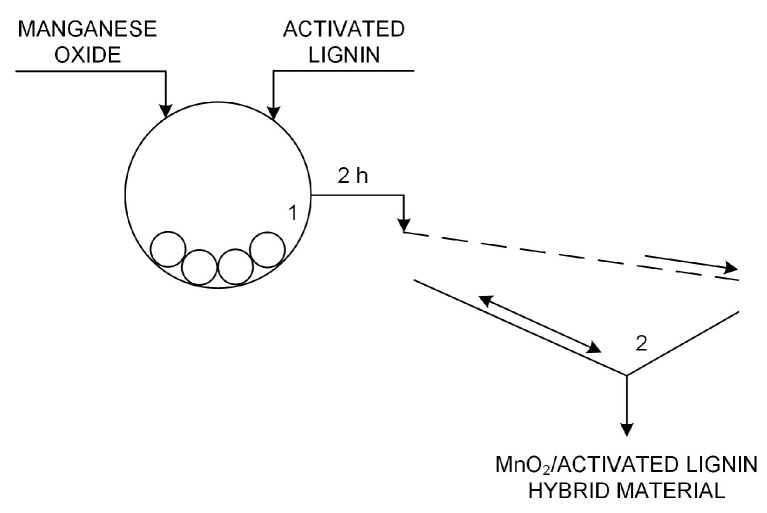
Technological scheme of preparation of manganese dioxide/activated lignin hybrid materials—simplified technological process (1—grinder mortar, 2—automatic sieve).

Activated lignins were used in the preparation of hybrid materials with manganese oxide (see Figure 19). To combine the MnO_2_ (Sigma-Aldrich, Hamburg, Germany) and lignin, a mechanical process was used whereby the initial powders were ground and simultaneously mixed using an RM 100 grinder mortar (Retsch GmbH & Co., Haan, Germany). To obtain three suitably homogeneous MnO_2_/lignin hybrid materials, grinding was continued for 2 h. To prevent possible overheating of the materials due to continuous grinding, every 30 min the mill automatically switched off for 2 min, after which it began operating again. Immediately after grinding, the inorganic-organic hybrid materials were sifted using a sieve with a mesh diameter of 40 μm. The hybrids obtained in this way then underwent further analysis and functional testing.

### 3.4. Characteristics of Materials

#### 3.4.1. Fourier Transform Infrared Spectroscopy

Fourier transform infrared spectra (FTIR) were recorded on a Vertex 70 spectrophotometer (Bruker AXS GmbH, Mannheim, Germany) and used to confirm the presence of the expected functional groups after the activation process in ionic liquids. Materials were analyzed in the form of tablets containing anhydrous KBr (ca. 0.25 g) and 2 mg of the tested activated lignins or hybrid materials, pressed between two steel rings under 10 MPa of pressure in a hydraulic press (SPECAC Ltd., Orpington, UK). Spectra were recorded in a wavenumber range of 4000–400 cm^−1^ (at a resolution of 0.5 cm^−1^).

#### 3.4.2. Elemental Analysis

The elemental comopsitions of activated lignins and MnO_2_/lignin hybrid materials were determined using the Vario El Cube system (Elementar Analysensysteme GmbH, Langenselbold, Germany). The analyzer is capable of registering the percentage content of carbon (C), hydrogen (H), nitrogen (N) and sulfur (S) in samples after high-temperature combustion. The results are given to ±0.01%.

#### 3.4.3. Scanning Electron Microscopy

The surface morphology and microstructure were examined on the basis of SEM images recorded from an EVO40 scanning electron microscope (Zeiss, Jena, Germany). The microscope accelerates electrons in the range 0.2–30 kV, providing a good resolution of the scanned material. Before testing, the samples were coated with Au for a time of 5 s using a Balzers PV205P coater (Oerlikon Balzers Coating, Brugg, Switzerland).

#### 3.4.4. Electrokinetic Properties

The zeta potential in the selected pH range was measured with a Zetasizer Nano ZS (Malvern Instruments Ltd., Worcester, UK) equipped with an MPT-2 autotitrator. The principle of measurement is based on electrophoresis and use of the Doppler effect of excited particle motion under laser light. The measurement was made by suspending 0.01 g of the tested sample in 25 mL of variable ionic strength electrolyte (0.001 M NaCl). The mixture was placed in an autotitrator vessel with continuous stirring and then titrated with a 0.2 M solution of HCl or NaOH. In the next step, a sample of known pH value was pumped into a measured cell equipped with an electrode. As a result of the application of variable electrical voltage, particle motion velocity was measured. The moving particles cause dispersion of laser light (λ = 633 nm), which is recorded by the detector. On this basis the electrophoretic mobility is determined, which is then converted to a zeta potential value.

#### 3.4.5. Thermal Analysis

The thermogravimetric analyzer TG 209 F3 Tarsus^®^ (Netzsch, Selb, Germany) was used to determine the thermal stability of the materials obtained. The assessment was conducted at temperatures in the range 25–600 °C, at 10 °C/min, under nitrogen flow. For TG analysis, 5 mg of the test sample was used. Thermogravimetric analysis is based on the recording of changes in mass of the material under controlled heating to the preset temperature, as illustrated graphically in the form of a thermogram. Differential thermal analysis makes it possible to identify the thermal effects associated with the physical or chemical changes of the test sample during heating. In practice it consists in simultaneous heating of samples and standard reference (Al_2_O_3_) and observation of the thermal transformations occurring.

#### 3.4.6. Porous Structure Properties

To determine the parameters of the porous structure of the activated lignins and MnO_2_/lignin hybrids, surface area, pore volume, and average pore size were determined using an ASAP 2020 apparatus (Micromeritics Instruments Co., Norcross, GA, USA). All samples were degassed at 80 °C for 4 h prior to measurement. The surface area was determined by the multipoint BET (Brunauer–Emmett–Teller) method. To determine the pore volume and average size the BJH (Barrett–Joyner–Halenda) algorithm was used.

### 3.5. Electrochemistry Research

#### 3.5.1. Materials

Graphite (G, SL-20, BET surface area 6.0 m^2^/g, Superior Graphite, Chicago, IL, USA), poly(vinylidene fluoride) (PVdF, M_W_ = 180,000 Fluka, Bucharest, Romania), lithium foil (Sigma-Aldrich, Hamburg, Germany, 0.75 mm thick), N-methyl-2-pyrrolidinone (NMP, Sigma-Aldrich, Hamburg, Germany), ethylene carbonate (EC, Sigma-Aldrich, Hamburg, Germany,), dimethyl carbonate (DMC, Sigma-Aldrich, Hamburg, Germany,) and lithium hexafluorophosphate (LiPF_6_, Sigma-Aldrich, Hamburg, Germany) were used. Solid LiPF_6_ salt was dissolved in the liquid solution: a classical electrolyte (1 M LiPF_6_ in EC + DMC, 1:1) was prepared in a dry argon atmosphere in a glove box. Tested anodes were prepared on a copper foil (Hohsen, Japan) by a casting technique, from a slurry of materials (M), graphite and PVdF in NMP. The ratio of components was AM:G:PVdF = 80:10:10 (by weight). The compositions of the electrodes are given in Table 8.

After vacuum evaporation of the solvent (NMP) at 120 °C, a layer of the carbon electrode was formed, containing the active material, an electronic conductor (G) and the binder (PVdF).

#### 3.5.2. Procedures and Measurements

Electrochemical properties of the cells were determined with the use of impedance spectroscopy (EIS) as well as tests based on galvanostatic charging/discharging. The ATLAS 0461 MBI multichannel electrochemical system (Atlas-Sollich, Rebiechowo, Poland) at different current rates was used for cycling measurements. Charging/discharging measurements were carried out at 20 °C. Cyclic voltammetry (CV) and AC impedance measurements were performed using the μAutolab FRA2 type III electrochemical system (Metrohm Autolab B.V., Utrecht, The Netherlands).

Electrodes were separated by the glass microfiber GF/A separator (Whatmann, Maidstone, UK, 0.4–0.6 mm thick), placed in an adapted Swagelok^®^ connecting tube. Typically, the mass of the electrodes was as follows: Li: ca. 45 mg (0.785 cm^2^), E: 3.5–4.0 mg. Cell assembly was performed in a glove box in a dry argon atmosphere.

After the electrochemical measurements, the cells were disassembled, and the composite electrodes were washed with DMC and dried in vacuum at room temperature. The morphology of the electrodes and polymer electrolytes was observed with a scanning electron microscope (EVO40, Zeiss, Jena, Germany). All operations were performed in a dry argon atmosphere in a glove box.

## 4. Conclusions

In this study, kraft lignin was activated using selected ionic liquids. The efficiency of the proposed method was confirmed by a series of analyses. FTIR analysis proved the efficacy of activation, especially by the recording of a band attributed to unconjugated carbonyl groups in lignin. The increase in the intensity of this band revealed that the activated biopolymer had more of these reactive groups. This is very important in the preparation of hybrid materials and their use as active materials in electrochemistry. Activation in ionic liquids had only a slight influence on the content of elements such as carbon, hydrogen, nitrogen and sulfur. However, pretreatment of lignin in ILs changed the morphology of the particles. They were smaller and had higher surface area, as confirmed by determination of the surface parameters. Characterization of surface functional groups was based on calculation of values of the zeta potential. A low value of the parameter confirmed the presence of dissociate groups such as hydroxyl, carboxyl and carbonyl groups. The thermal stability of the activated biopolymers was at a similar level to that of the pure product.

The activation of lignin in selected ionic liquids was completed by an effective process of IL regeneration. In the obtained spectra there were observed only bands attributed to hydroxyl groups (probably from water) and weak signals associated with aromatic rings.

These results provided encouragement to perform mechanical combination of manganese oxide with the activated lignins. The correctness of the synthesis was also confirmed based on the FTIR method. The obtained materials exhibited better electrokinetic, thermal and surface properties than the activated lignins. With regard to the low values of zeta potential, a full investigation was made of the potential utility of the materials in electrochemistry.

The MnO_2_/KL+B electrode could maintain a high discharge capacity of 665 mAh·g⁻^1^ over 20 cycles at a current density of 50 mA·g⁻^1^, and also exhibits a reversible capacity of 580 mAh·g⁻^1^ even at 1000 mA·g⁻^1^. Furthermore, the cycling performance for the MnO_2_/activated lignin composite is clearly much better than that of the bare MnO_2_.

The excellent electrochemical performance can be attributed to the unique structure of the composite, the high capacity of MnO_2_ and electrical conductivity of IL. Therefore, activated lignin based metal oxide composites is an effective method for improving on the electrochemical performance of the bare metal oxides as anode materials.

## Figures and Tables

**Table 1:** Vibrational wavenumbers (cm⁻^1^) for kraft lignin (KL).

Functional Groups	Vibrational Assignment	Frequency Wavenumbers (cm⁻^1^)	Appropriate Literature
–OH	stretching	3420–3390	[27,58,59]
–CH (CH_3_+CH_2_)	stretching	2934	[27,39,58,59,60,61]
–CH (OCH_3_)	stretching	2860–2840	[27,39,58,59,61]
–C=O (Unconjugated)	stretching	1715	[27,39,58,59,60,61]
–C–C– aromatic	aromatic skeletal	1597	[27,39,58,59,60,61]
–C–C– aromatic	aromatic skeletal	1506	[27,39,58,59,60,61]
C–H (CH_3_+CH_2_)	deformation asymmetric	1450	[27,39,58,59,60]
–C–C– aromatic	aromatic skeletal	1425	[27,39,58,59,60,61]
–OH phenolic	deformation	1370	[58,59,60,61]
C–O(G)	stretching	1266	[27,39,58,59,60,61]
C–O(H) + C–O(Ar)	stretching	1220	[39,58,59,60]
Ar C–H(G)	deformation	1150	[39,58,59,61]
Ar C–H(S)	deformation	1129	[27,39,58,59,61]
–CH in plane in guaiacyl and C–O in primary alcohols + ether	deformation	1032	[27,39,58,59,60,61]
–CH, –OH	banding	<900	[27,39,58,59,60,61]

**Table 2:** Elemental content of kraft lignin (KL) and lignin activated in selected ionic liquids: [C_4_im][HSO_4_] (KL+A), [Et_3_NH][HSO_4_] (KL+B), and [C_4_C_1_im][MeSO_3_] (KL+C).

Symbol	Elemental Content (%)			
	N	C	H	S
KL	-	62.3	6.7	3.9
KL+A	0.1	60.3	6.3	3.1
KL+B	0.1	59.4	6.5	3.2
KL+C	0.1	60.3	6.3	3.9

**Table 3:** Parameters of porous structure of pure kraft lignin (KL) and products activated in selected ionic liquids: [C_4_im][HSO_4_] (KL+A), [Et_3_NH][HSO_4_] (KL+B), and [C_4_C_1_im][MeSO_3_] (KL+C).

Symbol	Porous Structure Properties		
	*A_BET_* (m^2^/g)	*V_p_* (cm^3^/g)	*S_p_* (nm)
KL	1	0.01	12.1
KL+A	18	0.01	3.6
KL+B	14	0.01	3.8
KL+C	16	0.01	3.6

**Table 4:** Elemental contents of manganese oxide and MnO_2_/lignin hybrid materials based on activated biopolymer in selected ionic liquids.

Symbol	Element Content (%)			
	N	C	H	S
MnO_2_	-	0.1	0.5	-
MnO_2_/KL+A	0.2	30.2	3.4	1.6
MnO_2_/KL+B	0.2	29.5	3.3	1.5
MnO_2_/KL+C	0.2	30.3	3.3	2.0

**Table 5:** Parameters of porous structure of manganese oxide and MnO_2_/lignin hybrid materials based on biopolymer activated in selected ionic liquids.

Activating Agent	Porous Structure Properties		
	*A_BET_* (m^2^/g)	*V_p_* (cm^3^/g)	*S_p_* (nm)
MnO_2_	98	0.01	2.2
MnO_2_/KL+A	50	0.02	2.2
MnO_2_/KL+B	45	0.03	2.2
MnO_2_/KL+C	34	0.01	2.1

**Table 6:** The fitting value of the electrodes.

Electrode	Value (Ω)	MnO_2_	KL	MnO_2_/KL+A	MnO_2_/KL+B	MnO_2_/KL+C
BEFORE	*R*_SEI_	115	110	210	15	70
AFTER		75	50	160	8	25
BEFORE	*R*_ct_	190	168	374	31	135
AFTER		145	88	205	18	80

**Table 7:** Example of a functional anode materials.

Material	Characteristic Features	Reference Number
NiO@MnO_2_	Reversible capacity of 1573 mAh·g⁻^1^ is observed after 500 cycles at a current density of 0.53 A·g⁻^1^.	[124]
Li_2_-DMT	Material was characterized by a reduction potencial observed at 0.65V vs. Li. It displays a gravimetric capacity of 160 mAh·g⁻^1^ (128 mAh·g⁻^1^ by considering a composite electrode) even after 50 cycles associated with CE of 99.96%.	[125]
CPC—Coir pith derived carbon	Pristine CPC, upon investigation as anode in LIB applications exhibits a steady state progressive capacity of 837 mAh·g⁻^1^ at 100 mA·g⁻^1^ condition upon extended cycles.	[126]
MnO_2_ nanorods/3D-rGO	Electrochemical characterization exhibits the composite with large reversible capacity (595 mAh·g⁻^1^ over 60 cycles at 100 mA·g⁻^1^), high coulombic efficiency (above 99%).	[127]
Mn_3_O_4_/CNF	Mn_3_O_4_/CNF composites with 1.62%, 3.21% and 6.74% Mn, which exhibit enhanced reversible specific capacities of 486.1, 609.3 and 539.0 mAh·g⁻^1^, at 0.1 C after 100 cycles.	[128]
Mn_3_O_4_@C	The initial coulombic efficiency of this composite approach to 69.5%, and it delivers a capacity of nearly 420 mAh·g⁻^1^ even at a discharge current of 1800 mA·g⁻^1^.	[129]
N-TiO_2_@NC	At a current density of 5000 mA·g⁻^1^, the NTiO_2_@NC composites can deliver a reversible capacity of 232.7 mAh·g⁻^1^ after 2000 cycles.	[130]
TiO_2_-MnO_x_	The specific capacity of the composite stays above 972 mAh·g⁻^1^ for 100 cycles at a current rate of 100 mA·g⁻^1^.	[131]

**Table 8:** Ratios of components used to prepare electrodes (wt·%).

Electrodes (E)	Active Materials (AM) wt·%	G wt·%	PVdF wt·%
MnO_2_	80	10	10
KL			
MnO_2_/KL+A			
MnO_2_/KL+B			
MnO_2_/KL+C			
